# Supplementary material for: Transcriptomic response of brain tissue to focused ultrasound‐mediated blood–brain barrier disruption depends strongly on anesthesia
Source: Bioeng Transl Med. 2020 Nov 24;6(2):e10198. doi: 10.1002/btm2.10198 (PMC8126816; doi:10.1002/btm2.10198)
Supplement: Supplementary file 1 — Appendix S1: Supporting Information [file BTM2-6-e10198-s001.docx]

Supplementary Information for

**Transcriptomic Response of Brain Tissue to Focused Ultrasound-Mediated Blood-Brain Barrier Disruption Depends Strongly on Anesthesia**

A.S. Mathew^1^, C.M. Gorick^1^, E.A. Thim^1^, W.J. Garrison^1,3^, A.L. Klibanov^1,2^, G.W. Miller^1,3^, N.D. Sheybani^1,^*, and R.J. Price^1,3,^*

Richard J. Price

Email: rprice@virginia.edu

**
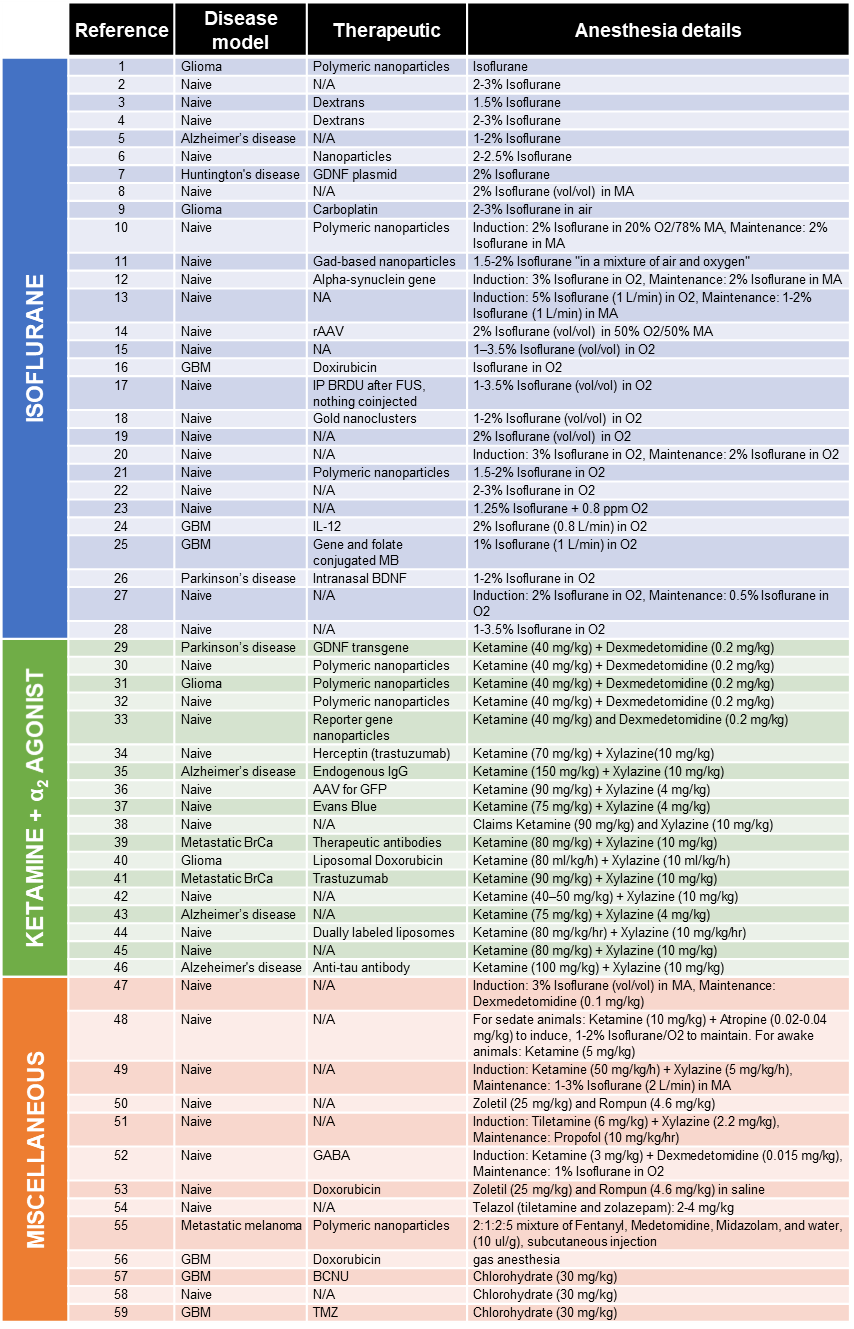
Table S1**

**Supplemental Figures**


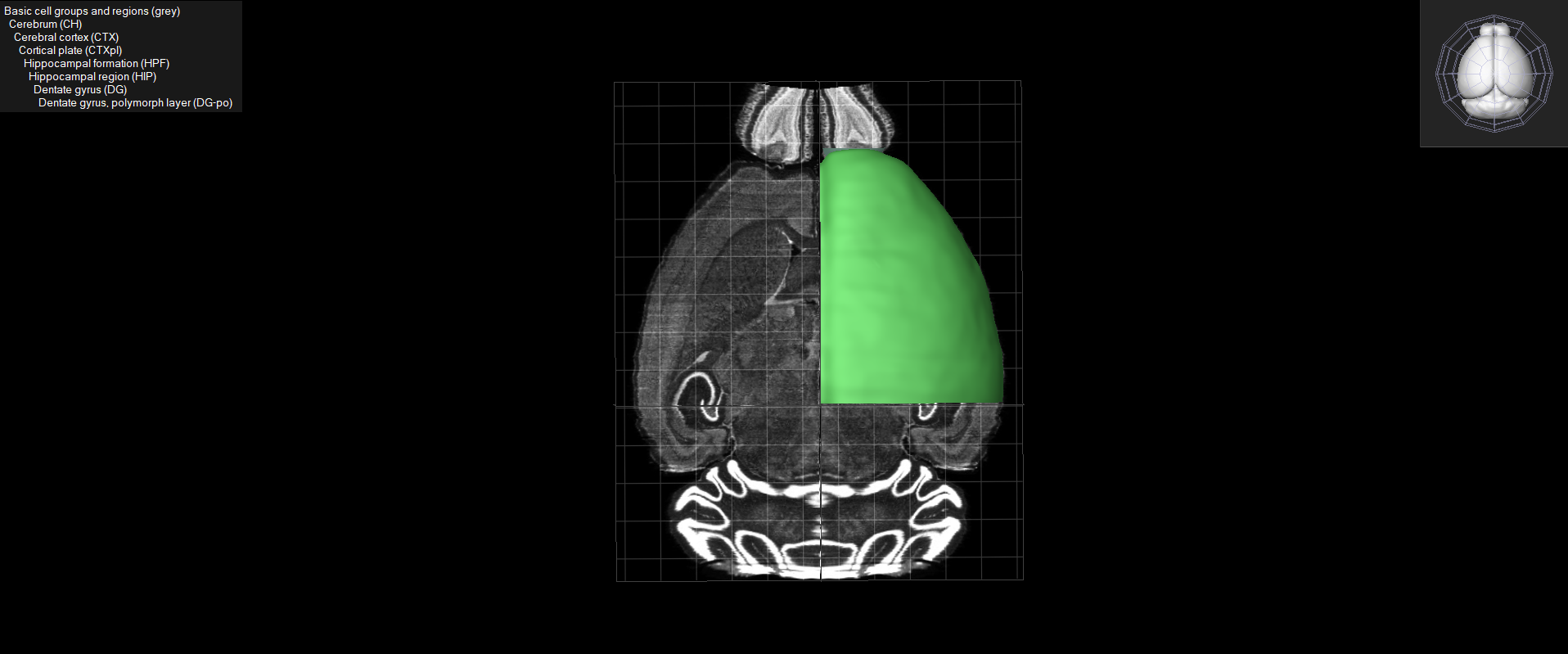


**Figure S1: Region of brain harvested for RNA-seq.** Top-down view of a 3-D rendering of a representative region of brain (green) harvested for RNA-sequencing, overlaid with an axial mouse MRI. Harvested regions weighed approximately 100 mg. The right quadrant was harvested for all sequenced mice, with the exception of one Iso-FUS mouse, which was treated on the left side.

**Figure S2: Voltage-Pressure calibrations for FUS transducers.** Voltage-Pressure calibrations for the two FUS systems used in this study. An MRIgFUS system (FUS Instruments RK-100, blue) was used for RNA-seq studies, while a tabletop system (Olympus A392S, red) was used for histological evaluation. Input voltages were determined for each system according to the calibration curve to achieve a matching output Peak-negative pressure (PNP) of 0.4 MPa (vertical dashed lines).

**References:**

1. R. J. Diaz, *et al.*, Focused ultrasound delivery of Raman nanoparticles across the blood-brain barrier: Potential for targeting experimental brain tumors. *Nanomedicine Nanotechnology, Biol. Med.* **10**, e1075–e1087 (2014).

2. B. Cheng, C. Bing, R. Chopra, The effect of transcranial focused ultrasound target location on the acoustic feedback control performance during blood-brain barrier opening with nanobubbles. *Sci. Rep.* **9**, 1–10 (2019).

3. M. A. Valdez, E. Fernandez, T. Matsunaga, R. P. Erickson, T. P. Trouard, Distribution and Diffusion of Macromolecule Delivery to the Brain via Focused Ultrasound using Magnetic Resonance and Multispectral Fluorescence Imaging. *Ultrasound Med. Biol.* **46**, 122–136 (2020).

4. D. Omata, *et al.*, Effects of encapsulated gas on stability of lipid-based microbubbles and ultrasound-triggered drug delivery. *J. Control. Release* **311**–**312**, 65–73 (2019).

5. M. E. Karakatsani, *et al.*, Unilateral focused ultrasound-induced blood-brain barrier opening reduces phosphorylated Tau from the rTg4510 mouse model. *Theranostics* **9**, 5396–5411 (2019).

6. J. Le Floc’h, *et al.*, Transcranial Photoacoustic Detection of Blood-Brain Barrier Disruption Following Focused Ultrasound-Mediated Nanoparticle Delivery. *Mol. Imaging Biol.* **22**, 324–334 (2019).

7. C. Y. Lin, *et al.*, Focused ultrasound-induced blood brain-barrier opening enhanced vascular permeability for GDNF delivery in Huntington’s disease mouse model. *Brain Stimul.* **12**, 1143–1150 (2019).

8. S. J. Mooney, J. N. Nobrega, A. J. Levitt, K. Hynynen, Antidepressant effects of focused ultrasound induced blood-brain-barrier opening. *Behav. Brain Res.* **342**, 57–61 (2018).

9. N. McDannold, *et al.*, Acoustic feedback enables safe and reliable carboplatin delivery across the blood-brain barrier with a clinical focused ultrasound system and improves survival in a rat glioma model. *Theranostics* **9**, 6284–6299 (2019).

10. A. K. O. Åslund, *et al.*, Nanoparticle delivery to the brain - By focused ultrasound and self-assembled nanoparticle-stabilized microbubbles. *J. Control. Release* **220**, 287–294 (2015).

11. A. Conti, *et al.*, Empirical and Theoretical Characterization of the Diffusion Process of Different Gadolinium-Based Nanoparticles within the Brain Tissue after Ultrasound-Induced Permeabilization of the Blood-Brain Barrier. *Contrast Media &#x26; Mol. Imaging* **2019**, 6341545 (2019).

12. C. J. Lin, *et al.*, Microbubble-facilitated ultrasound pulsation promotes direct α-synuclein gene delivery. *Biochem. Biophys. Res. Commun.* **517**, 77–83 (2019).

13. D. McMahon, K. Hynynen, Acute inflammatory response following increased blood-brain barrier permeability induced by focused ultrasound is dependent on microbubble dose. *Theranostics* **7**, 3989–4000 (2017).

14. Z. Noroozian, *et al.*, “MRI-guided focused ultrasound for targeted delivery of rAAV to the brain” in *Methods in Molecular Biology*, (Humana Press Inc., 2019), pp. 177–197.

15. Z. I. Kovacs, *et al.*, Disrupting the blood–brain barrier by focused ultrasound induces sterile inflammation. *Proc. Natl. Acad. Sci.* **114**, E75–E84 (2017).

16. F. Y. Yang, *et al.*, Focused ultrasound and interleukin-4 receptor-targeted liposomal doxorubicin for enhanced targeted drug delivery and antitumor effect in glioblastoma multiforme. *J. Control. Release* **160**, 652–658 (2012).

17. Z. I. Kovacs, *et al.*, MRI and histological evaluation of pulsed focused ultrasound and microbubbles treatment effects in the brain. *Theranostics* **8**, 4837–4855 (2018).

18. D. Sultan, *et al.*, Focused Ultrasound Enabled Trans-Blood Brain Barrier Delivery of Gold Nanoclusters: Effect of Surface Charges and Quantification Using Positron Emission Tomography. *Small* **14**, 1703115 (2018).

19. G. Samiotaki, *et al.*, Pharmacokinetic analysis and drug delivery efficiency of the focused ultrasound-induced blood-brain barrier opening in non-human primates. *Magn. Reson. Imaging* **37**, 273–281 (2017).

20. P. C. Chu, *et al.*, Focused Ultrasound-Induced Blood-Brain Barrier Opening: Association with Mechanical Index and Cavitation Index Analyzed by Dynamic Contrast-Enhanced Magnetic-Resonance Imaging. *Sci. Rep.* **6**, 1–13 (2016).

21. D. S. Hersh, *et al.*, MR-guided transcranial focused ultrasound safely enhances interstitial dispersion of large polymeric nanoparticles in the living brain. *PLoS One* **13** (2018).

22. C. Bing, *et al.*, Characterization of different bubble formulations for blood-brain barrier opening using a focused ultrasound system with acoustic feedback control. *Sci. Rep.* **8**, 1–12 (2018).

23. O. O. Olumolade, S. Wang, G. Samiotaki, E. E. Konofagou, Longitudinal Motor and Behavioral Assessment of Blood–Brain Barrier Opening with Transcranial Focused Ultrasound. *Ultrasound Med. Biol.* **42**, 2270–2282 (2016).

24. P. Y. Chen, *et al.*, Focused ultrasound-induced blood-brain barrier opening to enhance interleukin-12 delivery for brain tumor immunotherapy: A preclinical feasibility study. *J. Transl. Med.* **13**, 93 (2015).

25. C. H. Fan, *et al.*, Folate-conjugated gene-carrying microbubbles with focused ultrasound for concurrent blood-brain barrier opening and local gene delivery. *Biomaterials* **106**, 46–57 (2016).

26. R. Ji, *et al.*, Focused ultrasound enhanced intranasal delivery of brain derived neurotrophic factor produces neurorestorative effects in a Parkinson’s disease mouse model. *Sci. Rep.* **9** (2019).

27. Z. Cui, *et al.*, Enhanced neuronal activity in mouse motor cortex with microbubbles’ oscillations by transcranial focused ultrasound stimulation. *Ultrason. Sonochem.* **59**, 104745 (2019).

28. S. Sinharay, *et al.*, In vivo imaging of sterile microglial activation in rat brain after disrupting the blood-brain barrier with pulsed focused ultrasound: [18F]DPA-714 PET study. *J. Neuroinflammation* **16**, 155 (2019).

29. B. P. Mead, *et al.*, Novel Focused Ultrasound Gene Therapy Approach Noninvasively Restores Dopaminergic Neuron Function in a Rat Parkinson’s Disease Model. *Nano Lett.* **17**, 3533–3542 (2017).

30. E. Nance, *et al.*, Non-invasive delivery of stealth, brain-penetrating nanoparticles across the blood - Brain barrier using MRI-guided focused ultrasound. *J. Control. Release* **189**, 123–132 (2014).

31. K. F. Timbie, *et al.*, MR image-guided delivery of cisplatin-loaded brain-penetrating nanoparticles to invasive glioma with focused ultrasound. *J. Control. Release* **263**, 120–131 (2017).

32. B. P. Mead, *et al.*, Targeted gene transfer to the brain via the delivery of brain-penetrating DNA nanoparticles with focused ultrasound. *J. Control. Release* **223**, 109–117 (2016).

33. B. P. Mead, *et al.*, Focused Ultrasound Preconditioning for Augmented Nanoparticle Penetration and Efficacy in the Central Nervous System. *Small* **15**, 1903460 (2019).

34. M. Kinoshita, N. McDannold, F. A. Jolesz, K. Hynynen, Noninvasive localized delivery of Herceptin to the mouse brain by MRI-guided focused ultrasound-induced blood-brain barrier disruption. *Proc. Natl. Acad. Sci. U. S. A.* **103**, 11719–11723 (2006).

35. J. F. Jordão, *et al.*, Amyloid-β plaque reduction, endogenous antibody delivery and glial activation by brain-targeted, transcranial focused ultrasound. *Exp. Neurol.* **248**, 16–29 (2013).

36. M. Stavarache, N. Petersen, … E. J.-J. of, undefined 2018, Safe and stable noninvasive focal gene delivery to the mammalian brain following focused ultrasound. *thejns.org* (March 25, 2020).

37. J. Shin, *et al.*, Focused ultrasound-mediated noninvasive blood-brain barrier modulation: preclinical examination of efficacy and safety in various sonication parameters. *Neurosurg. Focus* **44**, E15 (2018).

38. N. McDannold, Y. Zhang, N. Vykhodtseva, Nonthermal ablation in the rat brain using focused ultrasound and an ultrasound contrast agent: long-term effects. *J. Neurosurg.* **125**, 1539–1548 (2016).

39. T. Kobus, I. K. Zervantonakis, Y. Zhang, N. J. McDannold, Growth inhibition in a brain metastasis model by antibody delivery using focused ultrasound-mediated blood-brain barrier disruption. *J. Control. Release* **238**, 281–288 (2016).

40. M. Aryal, N. Vykhodtseva, Y. Z. Zhang, J. Park, N. McDannold, Multiple treatments with liposomal doxorubicin and ultrasound-induced disruption of blood-tumor and blood-brain barriers improve outcomes in a rat glioma model. *J. Control. Release* **169**, 103–111 (2013).

41. E. J. Park, Y. Z. Zhang, N. Vykhodtseva, N. McDannold, Ultrasound-mediated blood-brain/blood-tumor barrier disruption improves outcomes with trastuzumab in a breast cancer brain metastasis model. *J. Control. Release* **163**, 277–284 (2012).

42. M. A. O’Reilly, K. Hynynen, Blood-brain barrier: Real-time feedback-controlled focused ultrasound disruption by using an acoustic emissions-based controller. *Radiology* **263**, 96–106 (2012).

43. J. Shin, *et al.*, Focused ultrasound-induced blood-brain barrier opening improves adult hippocampal neurogenesis and cognitive function in a cholinergic degeneration dementia rat model. *Alzheimer’s Res. Ther.* **11**, 110 (2019).

44. M. Aryal, *et al.*, MRI Monitoring and Quantification of Ultrasound-Mediated Delivery of Liposomes Dually Labeled with Gadolinium and Fluorophore through the Blood-Brain Barrier. *Ultrasound Med. Biol.* **45**, 1733–1742 (2019).

45. N. Todd, Y. Zhang, M. Livingstone, D. Borsook, N. McDannold, The neurovascular response is attenuated by focused ultrasound-mediated disruption of the blood-brain barrier. *Neuroimage* **201**, 116010 (2019).

46. P. W. Janowicz, G. Leinenga, J. Götz, R. M. Nisbet, Ultrasound-mediated blood-brain barrier opening enhances delivery of therapeutically relevant formats of a tau-specific antibody. *Sci. Rep.* **9**, 1–9 (2019).

47. P. C. Chu, *et al.*, Neuromodulation accompanying focused ultrasound-induced blood-brain barrier opening. *Sci. Rep.* **5**, 1–12 (2015).

48. S. Y. Wu, *et al.*, Efficient blood-brain barrier opening in primates with neuronavigation-guided ultrasound and real-time acoustic mapping. *Sci. Rep.* **8**, 1–11 (2018).

49. R. M. Jones, *et al.*, Three-dimensional transcranial microbubble imaging for guiding volumetric ultrasound-mediated blood-brain barrier opening. *Theranostics* **8**, 2909–2926 (2018).

50. H. Choi, E.-H. Lee, M. Han, S.-H. An, J. Park, Diminished Expression of P-glycoprotein Using Focused Ultrasound Is Associated With JNK-Dependent Signaling Pathway in Cerebral Blood Vessels. *Front. Neurosci.* **13**, 1350 (2019).

51. F. Prada, *et al.*, In vitro and in vivo characterization of a cranial window prosthesis for diagnostic and therapeutic cerebral ultrasound. *J. Neurosurg. JNS*, 1–13 (2020).

52. C. Constans, *et al.*, Non-invasive ultrasonic modulation of visual evoked response by GABA delivery through the blood brain barrier. *J. Control. Release* **318**, 223–231 (2020).

53. B. Jung, H. Huh, E. hee Lee, M. Han, J. Park, An advanced focused ultrasound protocol improves the blood-brain barrier permeability and doxorubicin delivery into the rat brain. *J. Control. Release* **315**, 55–64 (2019).

54. K. Yoon, *et al.*, Localized Blood–Brain Barrier Opening in Ovine Model Using Image-Guided Transcranial Focused Ultrasound. *Ultrasound Med. Biol.* **45**, 2391–2404 (2019).

55. H. Baghirov, *et al.*, Ultrasound-mediated delivery and distribution of polymeric nanoparticles in the normal brain parenchyma of a metastatic brain tumour model. *PLoS One* **13**, e0191102 (2018).

56. Z. Kovacs, *et al.*, Prolonged survival upon ultrasound-enhanced doxorubicin delivery in two syngenic glioblastoma mouse models. *J. Control. Release* **187**, 74–82 (2014).

57. H. L. Liu, *et al.*, Blood-brain barrier disruption with focused ultrasound enhances delivery of chemotherapeutic drugs for glioblastoma treatment. *Radiology* **255**, 415–425 (2010).

58. J. Xia, P. H. Tsui, H. L. Liu, Low-pressure burst-mode focused ultrasound wave reconstruction and mapping for blood-brain barrier opening: A preclinical examination. *Sci. Rep.* **6**, 1–11 (2016).

59. K. C. Wei, *et al.*, Focused Ultrasound-Induced Blood-Brain Barrier Opening to Enhance Temozolomide Delivery for Glioblastoma Treatment: A Preclinical Study. *PLoS One* **8** (2013).
